# Contribution of Shape Features to Intradiscal Pressure and Facets Contact Pressure in L4/L5 FSUs: An *In-Silico* Study

**DOI:** 10.1007/s10439-022-03072-2

**Published:** 2022-09-14

**Authors:** Amin Kassab-Bachi, Nishant Ravikumar, Ruth K. Wilcox, Alejandro F. Frangi, Zeike A. Taylor

**Affiliations:** 1 Institute of Medical and Biological Engineering (iMBE), School of Mechanical Engineering, University of Leeds, Leeds, LS2 9JT UK; 2 Centre for Computational Imaging & Simulation Technologies in Biomedicine (CISTIB), School of Computing, University of Leeds, Leeds, LS2 9BW UK; 3 Leeds Institute of Cardiovascular and Metabolic Medicine (LICAMM), School of Medicine, University of Leeds, Leeds, LS2 9JT UK

**Keywords:** Finite element models, Statistical shape models, Sensitivity analysis, Spine biomechanics, Virtual subjects

## Abstract

Finite element models (FEMs) of the spine commonly use a limited number of simplified geometries. Nevertheless, the geometric features of the spine are important in determining its FEM outcomes. The link between a spinal segment’s shape and its biomechanical response has been studied, but the co-variances of the shape features have been omitted. We used a principal component (PCA)-based statistical shape modelling (SSM) approach to investigate the contribution of shape features to the intradiscal pressure (IDP) and the facets contact pressure (FCP) in a cohort of synthetic L4/L5 functional spinal units under axial compression. We quantified the uncertainty in the FEM results, and the contribution of individual shape modes to these results. This parameterisation approach is able to capture the variability in the correlated anatomical features in a real population and sample plausible synthetic geometries. The first shape mode ($\phi _1$) explained 22.6% of the shape variation in the subject-specific cohort used to train the SSM, and had the largest correlation with, and contribution to IDP (17%) and FCP (11%). The largest geometric variation in ($\phi _1$) was in the annulus-nucleus ratio.

**Supplementary Information:**

The online version of this article contains supplementary material available 10.1007/s10439-022-03072-2.

## Introduction

We investigate the shape features that influence finite element model (FEM) results in a cohort of L4/L5 spinal segments. While spinal FEM studies commonly use simplified and/or limited geometries, the detailed anatomy at each spinal level is known to influence strongly the spine’s mechanical behaviour.^33,43^ In addition to improving the fundamental understanding of biomechanics of the lumbar spine, identifying the most influential shape features is important for identifying the anatomical features that should be captured with high fidelity during model construction. Various authors have explored this using FEM cohort-based studies.^3,29,32–34,43^ Such studies have been valuable in elucidating the biomechanical impact of inter-patient anatomical variability. However, two common and interlinked challenges in such investigations are: (1) effectively parameterising the relevant anatomical geometry so that geometric features may be investigated systematically; and (2) sampling the resulting geometry spaces so that the range of anatomical variability relevant to a target population is adequately covered. The second is to some extent a practical challenge, since both generating and solving FEMs for each sample may be computationally demanding. Geometry parameterisations with large numbers of parameters will obviously exacerbate this situation. The first challenge, on the other hand, is critical to ensuring meaningful geometric variations are represented. Moreover, it may ease or exacerbate the second by making generation of individual FEMs less or more difficult.

Having created a shape parameterisation, model instances may either be generated from real subjects, e.g., by extracting geometries from images and then fitting these to the shape model, or by randomly sampling parameter values and reconstructing the corresponding geometry. While the former guarantees that only realistic anatomies will be used, it is also limited by the availability of relevant image data. Such data, by definition, will also be biased towards anatomical averages, and will provide sparser samplings of anatomical extremes. Therefore, the latter approach has generally been favoured when investigating shape effects, and several automated spinal FEM generators have been developed.^5,6,23^ Lavecchia *et al.*^23^ generated full lumbar spine geometries from independently sampled geometric parameters. In contrast, Campbell *et al.*^5,6^ used a statistical shape modelling (SSM) approach, based on principal component analysis (PCA), for parameterising the shape of vertebral bodies in a group of full lumbar spines. They reported a specificity of 3.11mm and 3.76 mm at one and 17 shape modes, respectively, and a generalisation error of 3.65mm and 2.78mm at one and 16 shape modes, respectively.

Niemeyer *et al.*^33^ investigated the sensitivity of spinal FEMs to geometric variations in the L3/L4 segment using a comprehensive set of around 500 samples generated from a parametric geometry model. Their model consisted in an idealised representation of the vertebrae and disc geometries with 40 parameters associated with key anatomical features. Their investigation showed that geometry strongly influences the outcomes of FEMs of the lumbar spine. Zander *et al.*^43^ also showed that morphological features, as well as material properties, affect the outcomes of spinal FEMs using 1200 simulations based on a generic geometry of the full lumbar spine.Their model incorporated two geometric parameters representing the overall lordosis of the lumbar spine and the facet joint gap. Among other things, the findings of these studies highlight the limitations of employing averaged geometries or a small number of subject-specific geometries in spinal FE studies, as these cannot cover the full range of geometric variability present in a target cohort, rendering it difficult to generalise their conclusions to the underlying populations.

While previous studies successfully revealed the influence of various geometric features on spinal FEM predictions, the involved models contained no information about correlations between these features that are known to occur in real populations. That is, discrete features of spinal anatomies, such as distances between specific points, or areas/volumes of specific regions, do not vary independently of each other, but are correlated—often strongly so. By omitting these correlations, and instead allowing independent geometric feature variations, these parameterisation and sampling approaches likely produce geometries (or, specifically, combinations of geometric features) that, in practice, are not found in real cohorts.

Building on these previous works, and addressing the issue just described, we used a PCA-based SSM to parameterise the L4/L5 segment geometry.^9^ This model was subsequently sampled to generate realistic new geometries, which were used to assess the influence of naturally occurring shape variations on intradiscal pressure (IDP) and facet joint contact pressure (FCP) under pure axial compression. This method can produce segment samples that reflect the shape variability in a real cohort. Furthermore, although individual shape modes (SMs) are independent in the principal component space, each principal component (i.e., SM) will normally affect more than one anatomical feature in the Cartesian space. Correspondingly, this parameterisation approach is able, by construction, to capture the correlations between anatomical features in real populations and produce plausible new (sampled) geometries.

## Materials and Methods

**Figure 1:**
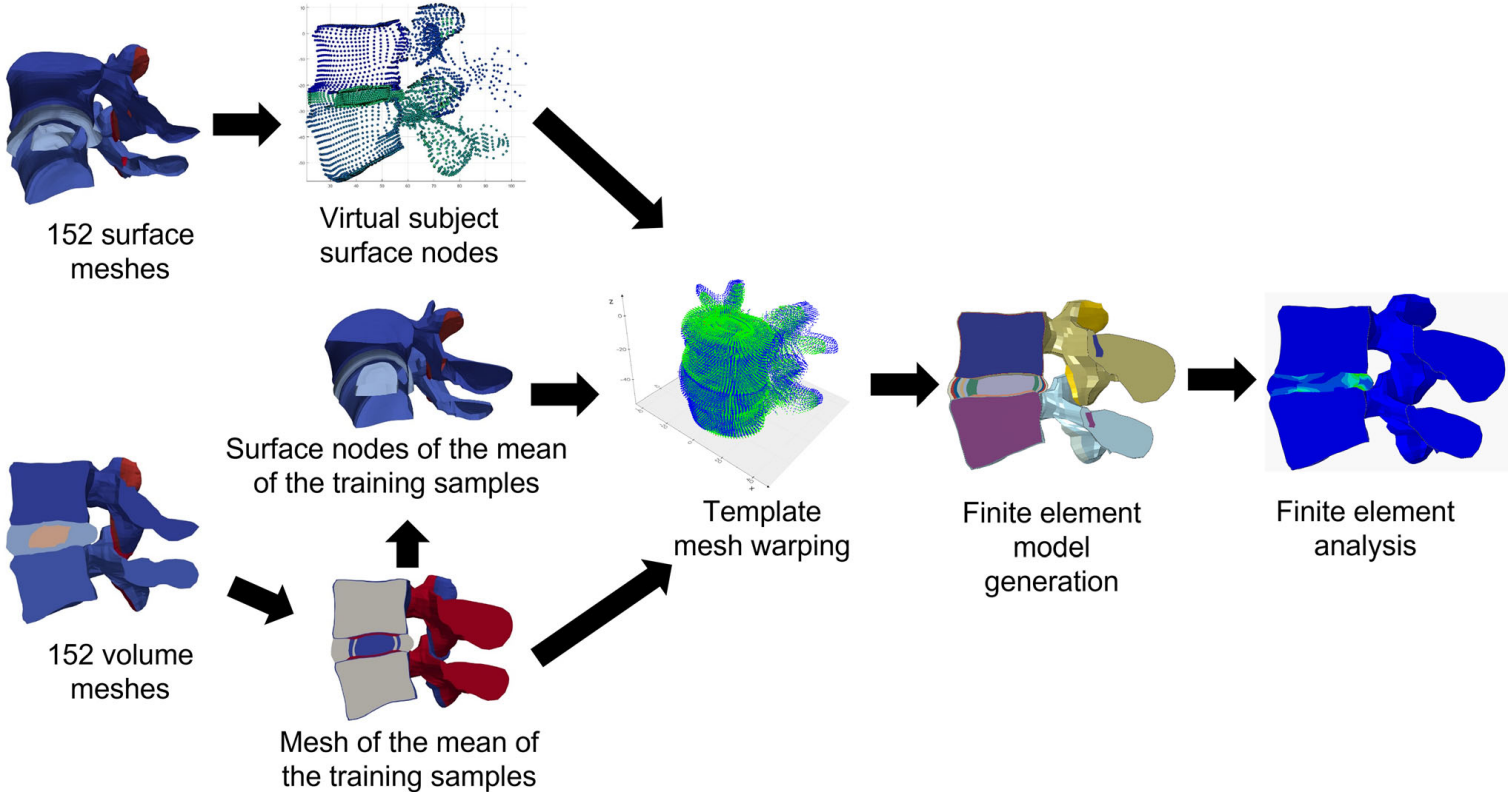
Workflow for acquiring FEA-based outcomes from synthetic L4/L5 FSUs generated using principal component-based statistical shape model.

The overall workflow for this study is shown in (Fig. 1) and each stage is described in more detail in the subsequent sections. Briefly, a dataset of 152 subject-specific L4/L5 functional spinal unit (FSU) geometries was used for two purposes, (1) develop a template FEM of the L4/L5 FSU and (2) train a principal component-based statistical shape model. The Latin hypercube method was used to generate 500 synthetic geometries from the SSM, meshed using a thin plate splines approach. Then, the FEM parameters were propagated from the template model to the synthetic geometries. The IDP and FCP outcomes of the training and synthetic FEMs were used for indirectly validating the FEM parameters. This workflow is shown in Fig. 1. Finally, the influence of shape features on the IDP and FCP in the synthetic subjects was evaluated through correlation and Shapley value analyses.

### Template L4/L5 FE Model

Abaqus/CAE 2017 (Dassault Systèmes Simulia Corp, US) was used for developing and analysing the FEMs of the L4/L5 segments.

#### Geometries

A cohort of 152 subject-specific, volumetric meshes of the L4/L5 FSU meshes were sourced from the MySpine project dataset.^7^ Each mesh was originally divided into regions representing the L4, L5, facet joints cartilage, nucleus pulposus and annulus fibrosus, including cartilaginous endplates (CEPs), as shown in Fig. 1 of the supplementary materials (Fig. 1S).^1^ The meshes had the same number of nodes with identical connectivity across subjects. Meshes were imported to Abaqus while preserving the anatomical structures’ original distinction. A single sample was chosen as a basis for the template FEM. Correspondingly numbered nodes and elements in different models represented the same anatomical structures, allowing the loads, BCs and material properties to be defined globally for specific numbered nodes and elements. Subsequently, these model parameters were propagated to the remaining 151 geometries using their corresponding node indices.

#### Materials

**Figure 2:**
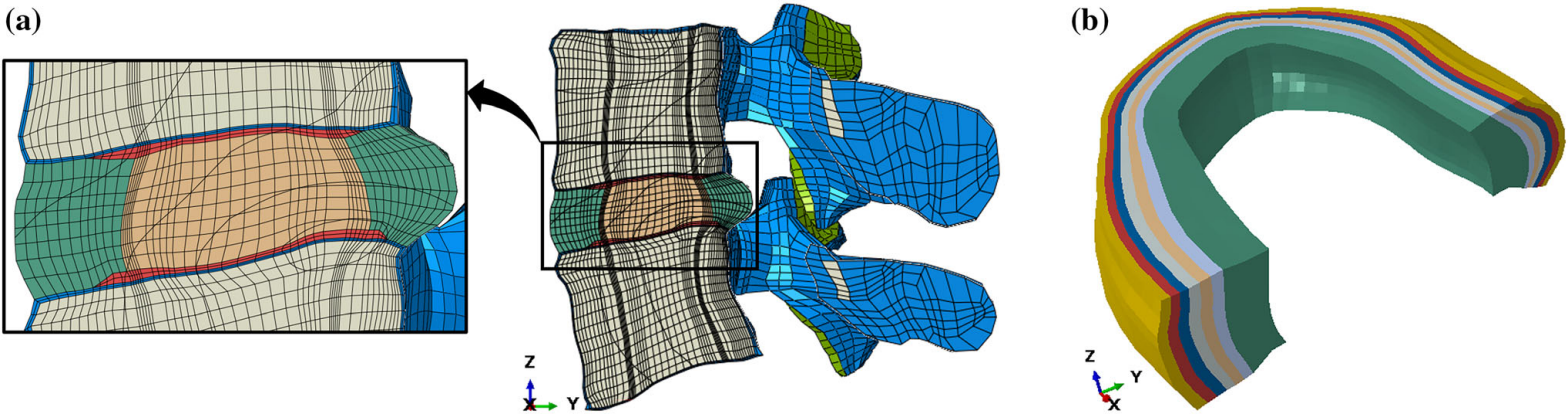
FEM materials definition in an exemplar L4/L5 spinal segment. (a) Cross sectional view, colour-coded according to different materials. (blue) Cortical bone, (beige) Cancellous bone, (orange) Nucleus, (red) CEPs, (dark green) Annulus, (light green) Facet joint cartilage. (b) cross sectional view of the annulus revealing the concentric layers.

The FSU was modelled using tri-linear solid hexahedral elements (Table 1). The mesh was divided into sections representing the material properties of different structures, based on regions defined in the original MySpine meshes (Table 2). Therefore, the vertebrae were divided into cancellous bone and cortical bone sections, with the cortical bone forming a two-element thick shell of variable thickness around the vertebral bodies. The upper and lower CEPs were sectioned from the top and bottom of the annulus region (Fig. 2a). The mesh between the CEPs, which represents the annulus fibrosus itself, was laterally divided into seven concentric layers (Fig. 2b).

The bony and cartilaginous structures of the vertebrae were modelled as linear elastic materials. The nucleus was modelled as a fully incompressible Neo-Hookean hyperelastic material. Holzapfel–Gasser–Ogden hyperelastic material model was used to model the fibre-embedded layers of the annulus fibrosus, with an alternating angle of approximately ${\pm }{30}^{\circ }$ fibre orientation, as shown in (Fig. 2S).

**Table 1:** Mesh details by structure.

Structure	Number of nodes	Number of elements	Element type
Cancellous bone	67,898	61,236	C3D8R
Cortical bone	35,612	22,360	C3D8R
Annulus fibrosus	8640	7040	C3D8
Nucleus Pulposus	8433	7168	C3D8RH
CEPs	7702	5184	C3D8R
Facets cartilage	1136	560	C3D8R
Total	108909^a^	103548	–

^a^Excluding duplicates of nodes shared between multiple structures

**Table 2:** Material parameters by structure.

Structure	Young’s modulus (MPa)	Poisson’s ratio			
Cancellous bone^38,44^	100	0.2			
Cortical bone^38,44^	12,000	0.3			
CEPs^27^	23	0.4			
Facets cartilage^37^	35	0.4			

	C$_{{10}}$ (MPa)	D (MPa$^{-1}$)	K$_{{1}}$ (MPa)	K$_{{2}}$	K
Annulus fibrosus^15^	0.34	0.306	1.8	11	0
Nucleus pulposus^30^	0.16	0.024	–	–	–

#### Loading and Boundary Conditions

**Figure 3:**
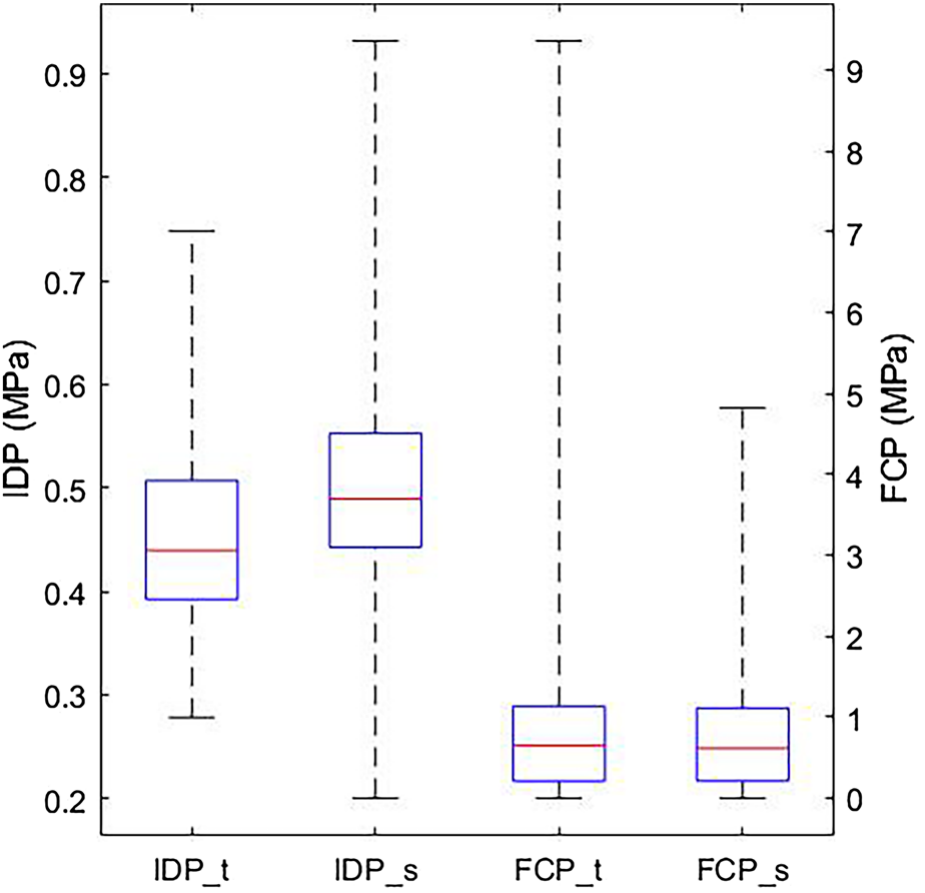
The FE outcomes of the training (IDP_t and FCP_t) and synthetic (IDP_s and FCP_s) models. The boxes represent the interquartile ranges, the red lines represent the medians and the whiskers show the range of values.

An axial load was applied to the upper bony endplate of the L4 vertebral body as a uniformly distributed pressure with a total resultant force of 400 N. The bottom bony endplate of the L5 was fixed entirely (Fig. 3S). All anatomically connected structures were modelled as fully bonded. Hard, frictionless contact was defined at the facet joint articulation surfaces.

#### Outputs and Validation

Several key outcomes are commonly measured when studying FSUs under different loading conditions. These include the range of motion in various directions, the facet joint forces or contact pressure and the intradiscal pressure. The intradiscal pressure (IDP) and the facets contact pressure (FCP) clearly reflect the FSU’s response to pure axial compression. Hence, they were chosen as key outcomes. The means of these outcomes, from the training and synthetic cohorts, were indirectly validated against values from previous *in-vivo* and computational studies performed under similar loading conditions.^12,22,26^

The IDP was measured as the maximum pressure within the nucleus pulposus. The FCP was measured as the maximum contact pressure over both the left and right facet joints of the L4/L5 segment. The means of these outcomes were calculated for the training and synthetic cohorts separately.

### PCA-based Statistical Shape Modelling

Ordered lists of 3-dimensional coordinates corresponding to *n* = 14,015 surface points from each of the training shapes (t=152) were used to train a statistical shape model, such that

1 $$\begin{aligned} {s}_{i} = \{{x}_{i1},\ldots , {x}_{p}, {y}_{i1},\ldots, {y}_{ip}, {z}_{i1},\ldots, {z}_{ip}\}, \end{aligned}$$

where $$ {s}_{i} $$ is the *i*th shape and *p* is the number of surface points. Surface points were taken from all exterior surfaces of the vertebrae and IVD, as well as internal boundaries at the annulus-nucleus and CEP-nucleus interfaces. The latter enables variations in the nucleus and CEP geometries, as distinct from the overall IVD geometry, to be captured. Other interior nodes from the volumetric meshes were omitted since their spatial positions do not correspond to real anatomical features (they were originally positioned so as to maximise finite element mesh quality); hence they do not carry meaningful anatomical information. The surface points from shapes {$$ {\mathbf{s}}_{{\text{2}}} , \ldots ,{\mathbf{s}}_{t}  $$} were aligned with the first sample $$\mathbf {s}_{1}$$. Size difference effects were also eliminated by applying rotations, translations and scaling through a Procrustes analysis.^13,18^ PCA was then performed, such that each shape ($$ {s}_{i}$$) can be represented as a sum of the mean shape ($${\bar{{s}}}$$) and a linear combination of shape modes ($$\phi _{m}$$) Eq. (2).

2 $$\begin{aligned}  {s}_{i} = {\bar{{s}}} + \sum _{m=1}^{M}{{w}_{m}\phi _{m}} \ \text {,} \end{aligned}$$

where ${w}_{m}$ is the weight associated with the *m*th shape mode, which represents its contribution to the shape change (i.e., deviation from the mean), and *M* is the total number of shape modes.

The model compactness with an increasing number of retained SMs was calculated as the cumulative sum of explained variance [3].

3 $$\begin{aligned} {C(M)}=\frac{1}{\lambda _{\text{total}}}\sum _{m=1}^{M}{\lambda _m}, \end{aligned}$$

where *C*(*M*) is the model compactness , $\lambda _m$ is the *m*th eigenvalue, $\lambda _{\text{total}}$ is the sum of all model eigenvalues and M is the total number of SMs produced by the model (i.e., 151 SMs).

The number of retained shape modes was chosen using a modified Kaiser’s rule,^21^ Eq. (4) which determines a minimum shape mode variance threshold such that,

4 $$\begin{aligned} \lambda _K = 0.7 \bar{\lambda } \ \text {,} \end{aligned}$$

where $\lambda _K$ is the shape mode variance threshold and $\bar{\lambda } = {1.0039\times 10^{3}}$ is the mean shape mode variance. Accordingly, the first 30 SMs were retained, explaining 86% of total shape variation. The effect of retaining more SMs on shape generation accuracy was also assessed at 41 and 64 SMs, explaining 90 and 95% shape variation, respectively.

The performance of the SSM was assessed in terms of accuracy, specificity, and generalisation ability. Accuracy estimates the model’s ability to regenerate the training samples by measuring the error between the training shapes and their corresponding shapes generated using the chosen subset of SMs. Specificity measures the model’s ability to generate shapes similar to the training shapes by calculating the error between statistically generated shapes and the most similar training shapes. The model’s generalisation ability measures its ability to generate new shapes of the same class as the training shapes. This was achieved using a leave-one-out cross- validation, where the SSM was trained using ($t-1$) of the training shapes, then applied to the remaining shape. The generalisation error is then calculated as an error between the original shape and its corresponding shape reconstructed using the chosen subset of SMs.

The average root mean squared error (*RMSE*) of the Euclidean distance between shapes was used to assess the different performance measures.

5 $$\begin{aligned} E_{i} = ||p_{i}-\hat{p_{i}}||_{2},\ {i} {= \{1,\ldots ,{n}\}} \ {,} \end{aligned}$$

6 $$\begin{aligned} {RMSE}_{s} = \sqrt{\frac{\sum _{i=1}^{n}{E_{i}^2}}{n}} \ \text {,} \end{aligned}$$

7 $$\begin{aligned} {RMSE} = \frac{\sum _{s=1}^{t}{{RMSE}_{s}}}{t} \ \text {,} \end{aligned}$$

where $E_{i}$ is the Euclidean distance between the *i*th corresponding points *p* and $\hat{p}$, ${RMSE}_{s}$ is the root mean squared error across all points for shape s, *RMSE* is the root mean squared error across shapes, *t* is the number of test shapes and *n* is the number of points in a shape.

For a better understanding of the generalisation ability of the SSM, we calculated the generalisation error with an increasing number of SMs.

### Synthetic Shapes Generation

A power analysis was conducted to calculate the sample size required for estimating the multiple correlations of 30 SMs with the chosen FEA outcomes. An exact test for linear multiple regression problems was employed in the G*Power package.^17^ For a study with 30 predictors, a medium detectable effect $\rho ^2=0.15$,^8,16^ a significance level $\alpha =0.05$ and a power $(1-\beta )=0.95$, the minimum required sample size was found to be 229 samples. Since around 40% of the FEMs failed in the preliminary tests due to element quality errors, it was decided that at least double the minimum calculated sample size must be used. Therefore, we generated 500 synthetic models.

We used the Latin Hypercube method^39^ to sample new instances from normally distributed SMs weights (${w}_{m}$) shown in Ref. [2]. Latin Hypercube is a popular method used for efficiently sampling high dimensional spaces by dividing each dimension into intervals equal to the number of required samples, then sampling a single point from each interval. It can be applied according to a specific criterion, such as maximising the minimum interval between the points or minimising their correlations. Since we are conducting a correlation study, we implemented the Latin Hypercube according to the correlations minimisation criterion.^19^ A $[500\times 30]$ matrix of ${w}_{m}$ was generated, where rows correspond to shapes and columns correspond to individual SMs. The weights ranged between $-2SD$ and $+2SD$ from the mean shape (i.e., $-2\sqrt{\lambda _{m}}$ and $+2\sqrt{\lambda _{m}}$).

### Meshing

As mentioned, each of the meshes from the MySpine dataset contained the same number of nodes and shared a common element connectivity. This common size and connectivity were also carried over to the SSM-generated synthetic subjects. However, the SSM generates new positions for surface nodes only (including those on internal anatomical boundaries, as described). Hence, a procedure is needed for re-positioning the remaining interior nodes to reflect the new positions of surface nodes. This mesh morphing was done using 3-dimensional thin plate splines (TPS)^4^ as implemented within the vedo library.^31^ The positions of corresponding surface nodes on the SSM-generated models and a template mesh were used as target and source points, respectively, to drive the TPS volumetric deformation. This deformation was then applied to all other interior nodes.

To create the template mesh, the nodes of each training model (i.e., each MySpine model, containing 108,909 nodes) were aligned by applying the transformation matrices from the earlier Procrustes analysis. As described, the Procrustes analysis was performed only on the extracted surface nodes; the resulting transformations were now applied on all nodes. The mean volumetric geometry (node positions) was calculated and, with the original connectivity also carried over, used as the template. Ideally, all synthetic subjects’ surface points and corresponding template surface points would serve as control points. However, the TPS algorithm is computationally demanding, and using all surface points leads to prohibitive processing times. Hence, a random sample of 1000 surface points (from 14,015 total) was used. The same 1000 points were used throughout for consistency across all synthetic subjects.

### Outcomes Uncertainty Quantification

FEM outcomes are considered as random variables associated with a certain level of uncertainty in evaluation. We quantified the uncertainty of the FE outcomes for the synthetic models by calculating the 95% confidence interval of the outcomes’ means estimations using a bootstrap sampling method with 1000 iterations. Bootstrapping creates new samples by repeatedly and randomly sampling from the original sample (i.e., the synthetic model results) with replacement. 1000 bootstrap iterations create 1000 new samples, otherwise known as bootstrap samples. Each sample has the same size as the original sample from which they were created, but they do not necessarily contain all the original sample members. This leads to dispersion of the outcomes around a mean. Calculating the confidence interval of this dispersion quantifies the uncertainty in the outcomes of the original sample (i.e., synthetic models) by estimating the limits within which the outcomes are expected to vary in the underlying population from which the original sample was taken. We used bootstrap sampling with 1000 iterations with gradually increasing sample sizes to gain a better understanding of the influence of sample size on the uncertainty of our FE outcomes.

### Shape Mode Importance Analysis

Levels of correlation between IDP or FCP and individual SMs were assessed through Pearson’s linear correlation, and Spearman’s ranked correlation. Both correlation measures were used to investigate the linearity in input-output relationships. Global sensitivity was analysed by estimating Shapley values using the SHAP kernel.^28^ Shapley analysis, theoretically, considers all possible combinations (i.e., coalitions) of predictors to assess individual predictor contributions to the deviation of an outcome from the fitted mean. Considering all the possible combinations of 30 parameters is impractical. Therefore, the algorithm default of 1,024 most influential combinations were considered.^28^ Normalised averaged absolute Shapley values showed the percentage of contribution of each shape mode to IDP and FCP, and the most influential SMs were deemed as “important” in predicting the outcomes.

## Results

### Indirect FE Model Validation

The training models had a mean IDP of 0.45 MPa, which is reasonably close to the computational and experimental value found in literature.^12,22^ The mean FCP of 0.88 MPa was reasonably close to the experimental result reported by Lorenz *et al.*^26^ under similar loading magnitude. Also, FCP values within the interquartile range (Fig. 3) were close and generally smaller than values reported under extension, axial torsion, and lateral bending,^22^ which is expected for the pure axial compression case. Keeping in mind that these results are only valid for the specific models included in this study, they were considered an indication of the training models producing the expected results.

The synthetic models had mean IDP and FCP of 0.51 MPa and 0.77 MPa, respectively. This indicated a good agreement with values from the literature and the training models. As shown in (Fig. 3), the training and synthetic models’ results were also similar in terms of their interquartile ranges and the location of the medians within the interquartile ranges. On the other hand, there was a clear difference between the two cohorts in terms of the range of extreme (i.e., minimum and maximum) values. It may be worth noting that while the range of IDP in the synthetic cohort increased, the range of FCP values decreased.

### Statistical Shape Model

**Figure 4:**
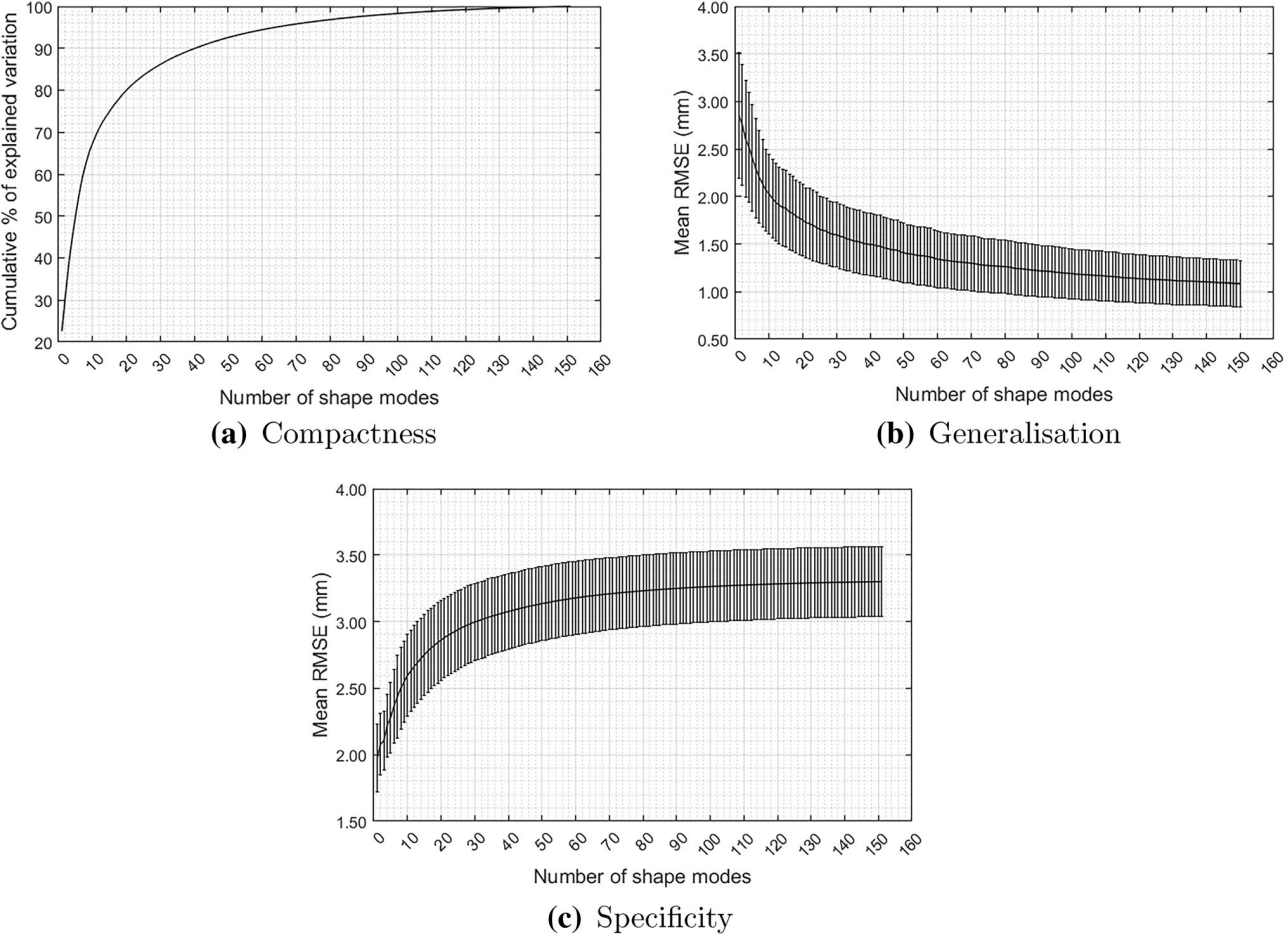
SSM performance change with number of retained SMs. (a) Model compactness, (b) model generalisation error, (c) specificity error.

Kaiser’s Rule showed that 30 SMs should be sufficient for representing the shapes. The 30 SMs explained  86% of the shape variation while increasing the explained variation to 90%, or 95% required 41 and 64 SMs, respectively, as shown in (Fig. 4a). The generalisation error was expressed as the average RMSE of all test samples reconstructed with an increasing number of SMs [7]. The generalisation error curve begins to level at around the 30 SMs mark (Fig. 4b). The model’s compactness, accuracy, specificity and generalisation ability are presented in (Table 3).

**Table 3:** SSM performance measures.

Shape modes	Explained shape variation	Accuracy (RMSE)	Specificity (RMSE)	Generalisation (RMSE)
30	86%	1.22 ${\pm }{0.12}$ mm	3.00 ${\pm }{0.29}$ mm	1.60 ${\pm }{0.34}$ mm

### Uncertainty Quantification

**Figure 5:**
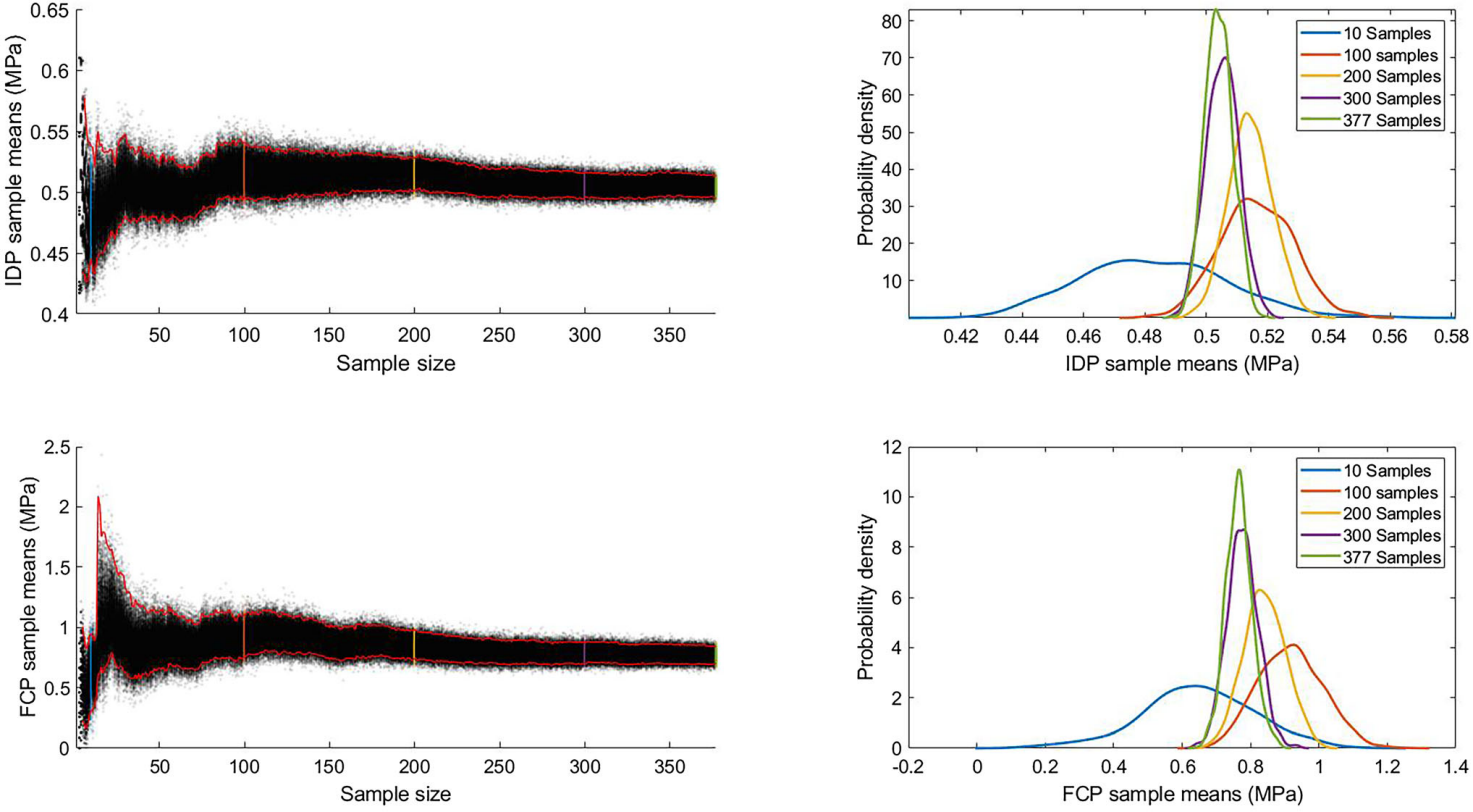
Bootstrapped sample means distribution and 95% confidence interval bounds (red curves) over sample size, with darker dots representing higher probability densities (left). Corresponding probability distribution of sample means at various sample sizes (right).

Plotting the synthetic FEMs sample means against the sample size showed that the confidence interval (CI) bounds decreased steadily with the increase in sample size (Fig. 5). For both FE outcomes, this decrease in the CI bounds become very small, starting at around 300 samples. Estimating the sample means probability density at multiple sample sizes also shows that the sample means probability densities converge towards specific values. At 377 bootstrapped samples, the estimated maximum sample means probabilities were at 0.504 (CI [0.495–0.512]) MPa and 0.76 (CI [0.69–0.86]) MPa for IDP and FCP, respectively. Unlike the training FEMs, FCP showed higher variability than IDP. The FCP is associated with a higher uncertainty, with its confidence interval an order of magnitude larger than that of the IDP.

### Shape Mode Importance

**Figure 6:**
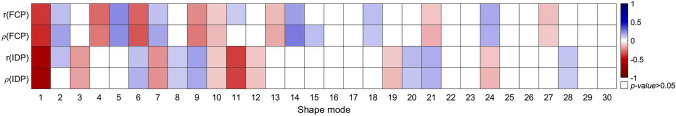
Individual shape mode correlations coefficients and significance levels. r and $\rho$ are Pearson’s and Spearman’s correlation coefficients, respectively. White cells represent no correlations, or insignificant correlations with *p-value* $>0.05$.

We found generally weak correlations between the SMs and the FE outcomes using Pearson’s and Spearman’s correlations (Fig. 6). Nevertheless, 15 of the correlations were considered significant at 0.05 significance level, except Spearman’s correlation for the IDP, where 14 SM correlations were considered significant. The two correlation methods had very close results in terms of levels and significance of correlation. Therefore, the relationship between SM and the FE outcomes was assumed to be close to linear. Performing sensitivity analysis based on a linear regression model was, therefore, appropriate.

**Figure 7:**
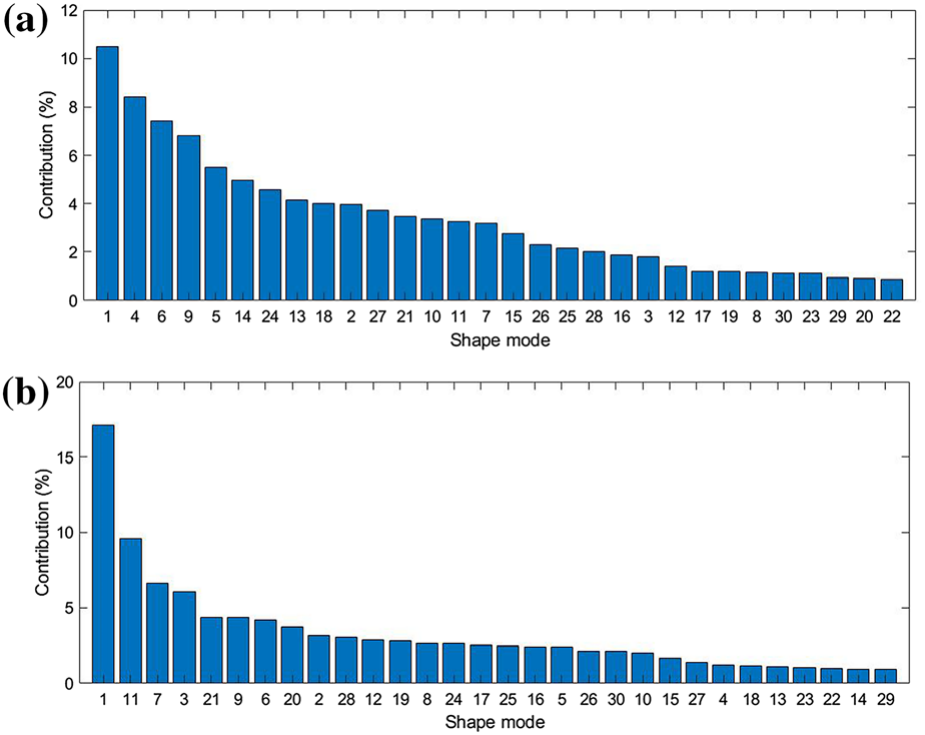
Individual shape mode percentage of contribution to IDP (a) and FCP (b).

The first SM ($\phi _{1}$) was found to contribute the most to both the IDP and FCP (Fig. 7). In the case of IDP, $\phi _{1}$’s contribution is considerably larger than the rest. In contrast, FCP shows a more distributed dependence on more shape modes. For both outcomes, the vast majority of SMs had less than a $5\%$ contribution. A relatively larger drop in contribution percentage was noticed after the first four most influential contributors, as can be seen in (Fig. 7). For simplicity, this relatively larger drop in contribution was considered a reasonable cutoff for considering an SM as “important” for visualisation and discussion.

**Figure 8:**
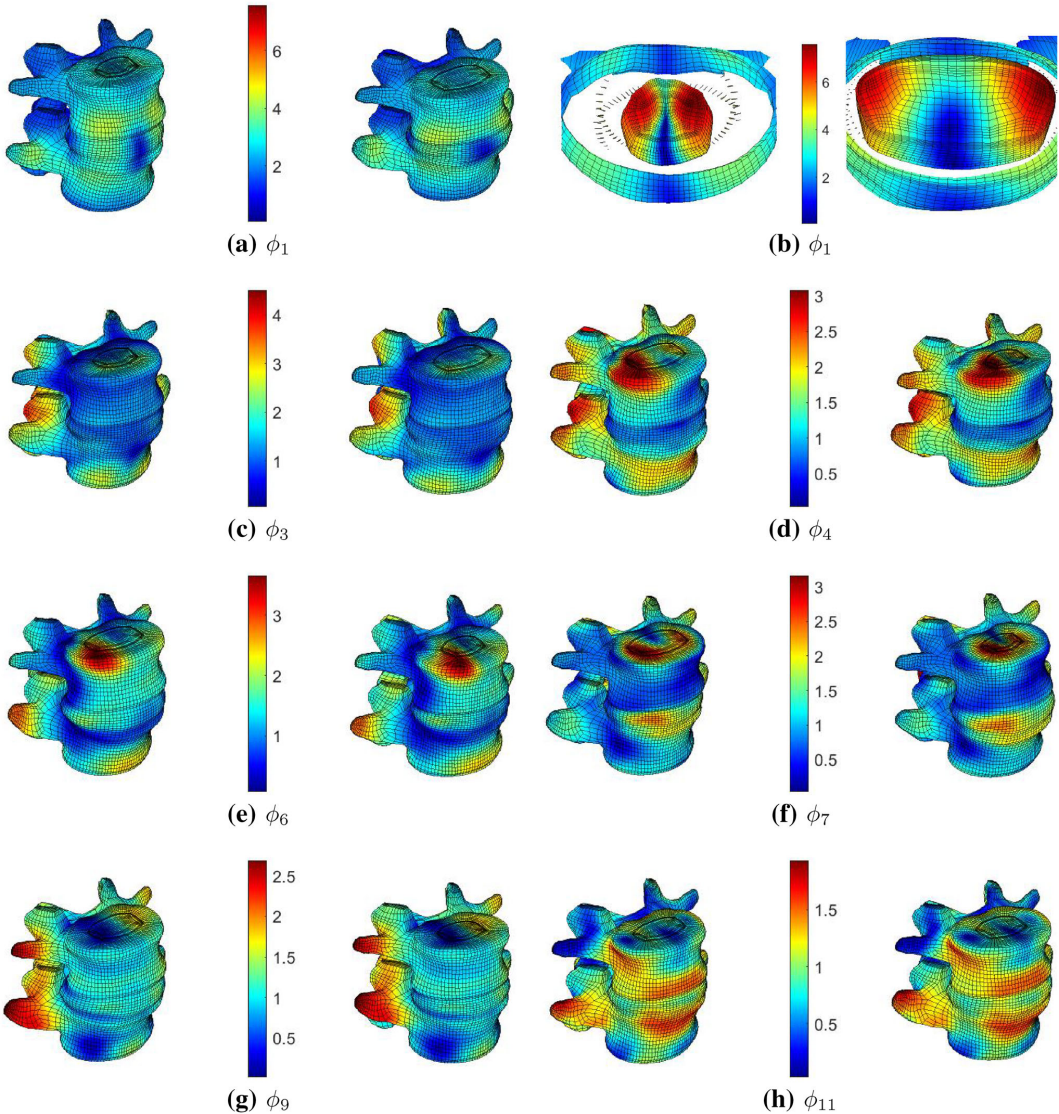
Visualisation of the seven most important SMs (i.e., one common to IDP and FCP, and three others specific to each outcome—(cf. Table 4)). The heat map represents the Euclidean distance from the mean shape in (mm) at $-2SD$ (left of colour bar) and $+2SD$ (right of colour bar) for $\phi _{1}$ (a,b), $\phi _{3}$ (c), $\phi _{4}$ (d), $\phi _{6}$ (e), $\phi _{7}$ (f), $\phi _{9}$ (g) and $\phi _{11}$ (h). The largest geometric effect in $\phi _{1}$ is on the size of the nucleus, and for this reason the nucleus is separately depicted in (b).

An SSM represents shape variation in a very efficient way. Where complex variations are involved, describing or even visualising the details of such variations becomes difficult. Figure 8 compares the mean shape to shapes at ${\pm }2SD$ of each important shape mode, and Table 4 briefly explains their most significant morphological effects (i.e., patterns of shape variation) noticed.

**Table 4:** morphological effects of important SMs.

Shape mode number	Shape variation explained (%)	Relevant outcome (importance rank)	Morphological effects ($+2SD$) from mean shape
$\phi _{1}$	22.6	IDP (1), FCP (1)	A general decrease in spinal segment height with an increase in the circumferential size. A large increase in the nucleus pulposus size in the lateral and posterior directions, with a slight decrease in the anterior nucleus pulposus thickness. This increased the IVD bulge in the posterior direction in particular
$\phi _{11}$	1.9	IDP (2)	An increase in the circumferential size of the vertebral bodies and IVD, and a slight decrease in the segment height. This resulted from an increase in the nucleus height and a decrease in the vertebral bodies’ heights. In addition to the increase in height, the nucleus increases in size in the anteroposterior direction and slightly decreases laterally
$\phi _{7}$	4.2	IDP (3)	A large increase in the anterior IVD bulge due to an anterior increase in the nucleus pulposus size. This is associated with a decrease in the posterior IVD bulge. The vertebral bodies increase in circumferential size and become more skewed in the frontal plane
$\phi _{3}$	7.7	IDP (4)	An Increase in the circumferential size of the vertebral bodies and IVD, especially in the bottom anterior region of the L5. A general, almost uniform, increase in the nucleus size. A large change in the facet joints’ orientation as they become oriented more posteriorly
$\phi _{4}$	6.1	FCP (2)	A large change in the relative anteroposterior positions of L4 and L5. The segment also exhibits an overall lordosis increase. The nucleus shows a general decrease in height, with an increase in anterior circumferential size. A large decrease in the facets clearance is noticed. Contact surfaces extend inferiorly on the L4 side and posteriorly on the L5 side. Increasing the facet joint area
$\phi _{6}$	4.9	FCP (3)	A decrease in segment lordosis associated with a decrease in the anterior nucleus height
$\phi _{9}$	2.9	FCP (4)	Larger facet joints, especially on the right side. It also tightens the clearance between contact surfaces. The frontal alignment of the two vertebrae is also affected

The first shape mode ($\phi _{1}$), which explains $22.6\%$ of the shape variation in the training dataset, was the largest contributor for both the IDP ($\approx {17}\%$) and FCP ($\approx {11}\%$). It also produced the largest shape change in terms of Euclidean distance. This perhaps explains $\phi _{1}$ being the largest contributor to the outputs. The largest effect it had, as described in (Table 4), was on the nucleus. This is shown in (Fig. 8b).

It may also be worth noting that the main morphological effects due to each important SM produced the expected effect on each relevant FEA outcome. For example, a higher weight of $\phi _{1}$ increases the size of the nucleus and is negatively correlated to IDP. Similarly, a higher $\phi _{4}$ weight produces larger facet joint contact surfaces and is negatively correlated to FCP. These results seem sound in terms of the expected effect on mechanical behaviour.

## Discussion

The statistical shape modelling approach facilitates an efficient generation and analysis of virtual geometries of the L4/L5 spinal segment. It naturally allowed the most dominant shape variations in the training data to emerge from the dataset. Therefore, efficiently presenting the most relevant (i.e., most dominant and most important) shape features holistically. While discrete geometric features (e.g., intervertebral disc height, facet joints contact clearance, etc.) may be useful in some contexts, they are largely inaccurate in describing the realistic shape changes presented here. That is because shape features do not change rigidly. For instance, the disc height might increase greatly in the central region of the disc while the height around the edges of the disc stays constant. Furthermore, independently sampled discrete geometric features are likely to produce feature combinations that do not occur in real populations. In contrast, the statistical shape modelling approach provides insight into the natural co-variations between different geometric features. For example, a higher $\phi _{11}$ weight results in a decrease in overall segment height is expected when the anterior regions of the segment (i.e., vertebral bodies and IVD) have larger diameters. A limitation of this approach is its inability to explore anatomical extremes beyond the range of the training shapes.

Furthermore, PCA, as a linear approach, is limited in its ability to model the rigid angular motions between articulating structures. Visual inspection of the full lumbar spine geometries from which our samples were extracted suggested no significant inter-subject postural variability (i.e., underlying images were acquired in a neutral spine position). Nevertheless, it is inevitable that some variability remains. Such postural differences may affect the fidelity of some shape features that are directly impacted by the relative positions of the vertebrae, such as the segment lordosis or the facets’ contact clearance, while other features, such as the annulus-nucleus ratio and the facet joint articulation surface area are not likely to be affected. Also, it is assumed when using PCA that the training data are normally distributed. A deviation from this assumption results in non-linear correlations between principal components, which may affect the SSM’s specificity and compactness. It has been suggested that nonlinear models, such as kernel PCA, principal polynomial shape analysis and principal geodesic analysis can overcome these limitations, possibly leading to models with improved specificity and compactness, capable of capturing complex deformation patterns better than linear methods such as PCA.^1,14,42^ Use of such non-linear shape parameterisation approaches in assessing the FE models’ sensitivity is an interesting future direction.

While size change is commonly the primary source of shape variation in a given biological cohort,^2,21^ which may, unintentionally, diminish the contribution of more subtle shape features that distinguish subjects more objectively in a given target cohort. Therefore, eliminating any size (i.e., scale) mismatch between training samples during Procrustes registration is expected to produce a more effective sensitivity analysis. Nevertheless, the shapes generated from the statistical shape model will all have similar sizes. The similarity between FE results from the training and synthetic cohorts indicates that the effect of minimising size variation in the synthetic cohort on FEA outcomes was not great. Nonetheless, it may partially explain the observed difference in the range of values shown in (Fig. 3).

The 30 retained SMs produced shapes with reasonable overall accuracy. During accuracy analysis, the local error patterns in individual training geometries that were reconstructed using the retained SMs showed lower local accuracy in the posterior region of the spine. This was mainly around the posterior aspects, the superior and inferior facets in the L4 and L5, respectively, and at the root of the lateral aspects. Nonetheless, these structures were still plausible representations of the training geometries. Furthermore, the affected regions are not expected to significantly impact the FEA or the importance results presented in this work. Therefore, it was decided that the amount of error was acceptable for the application.

The large decrease in the sample mean confidence intervals until around 300 samples suggests that the small sample sizes commonly used in spinal FE studies, whether of simplified or realistic FSU geometries, would have substantial uncertainty in representing the underlying cohort from which the FSUs were sampled. Furthermore, in addition to the spatially variable material properties, studies suggest a large inter-subject variation in material properties of the spine.^10^ Therefore, the uncertainty is expected to increase further with the inclusion of more complexities, such as material properties that vary spatially within a subject and/or across subjects.

FCP exhibited substantially larger uncertainty than IDP. Because only a small decrease in the confidence intervals for both outcomes was seen after the 300 samples mark, increasing the sample size is unlikely to greatly improve the FCP certainty. This is expected, partly due to its sensitivity to the shape of the contact surfaces. Capturing such details of the facet joint cartilage, such as its thickness, using an imaging system is exceedingly difficult. Therefore, shape variations relevant to these details, such as the facets contact clearance, can be considered as rough approximations. More importantly, FCP is highly mesh dependant, because its value will depend on the location of the actual maximum contact pressure with respect to the node where the maximum value was calculated, which adds another level of randomness that contributes to the uncertainty of its results. Mesh dependence of the FCP could have been minimised through a mesh independence study. However, this was not possible with the mesh generation process used here. This is indeed expected to have an impact on the accuracy of FEMs and, consequently, on the sensitivity analysis for both outcomes, but more so for the FCP.

Pearson’s and Spearman’s correlation coefficients were similar. This showed that the relationship between the SMs and either FE outcomes is close to linear. Therefore, it was appropriate to base the Shapley analysis on a linear regression system. Sensitivity analysis showed that both the IDP and the FCP are dominantly sensitive to the first SM ($\phi _{1}$). Also, IDP is more sensitive to $\phi _{1}$ than FCP. In (Table 4), it was noted that the first SM predominantly affects the width of the nucleus pulposus. This effectively changes the nucleus-annulus ratio in the IVD. This shows that this aspect of the geometry is highly variable in the dataset and that both the IDP and FCP are highly sensitive to it. The number of considered predictor combinations, also called feature subsets, in the Shapley analysis was relatively small compared to the number of possible combinations (i.e., $2^{30}$). The impact of this on the sensitivity analysis was not directly determined in this study. Nonetheless, The SHAP algorithm used calculates the weight of each subset based on its cardinality (i.e., the number of predictors in the subset), where subsets with very high and very low cardinality values have higher weights.^28^ The algorithm includes predictor combinations in descending order of weights. Therefore, minimising the effect of not using all possible combinations.

While the results of this subject-specific study may only apply to the specific samples involved in this analysis, it showed that using PCA-based synthetic spinal cohorts can provide valuable insight into the mechanical responses in a target population and their sensitivity to specific shape features. A more anatomically objective representation of the samples provided a better understanding of the natural and complex anatomical combinations on the FE outcomes.The results suggest that such an anatomical objectiveness plays an important role in understanding the mechanical behaviour of the FSU in a more realistic sense. For instance, Niemeyer *et al.*^33^ found that the IVD height is the most important predictor of IDP. However, although disc height was observed in one of the important SMs, it is shown here that disc height had much less influence on the IDP value than the annulus-nucleus ratio. Also, as mentioned in (Table 4), the weight of $\phi _{9}$ is directly proportional to the clearance between facet joints contact surfaces. FCP is expected to be directly proportional to facets contact clearance.^33^ Nonetheless, results here (Fig. 6) show that FCP is negatively correlated with $\phi _{9}$. These differences can be explained, at least in part, by the different combinations of anatomical features that emerged from the training data at hand. A clear example of the effect of the co-variation of shape features is noticed with $\phi _{4}$, where a larger $w_{4}$ clearly decreases the facets clearance, which is expected to increase the FCP. Nonetheless, a larger $w_{4}$ greatly increases the facets contact surfaces area, as well as changes their angles, eventually leading to a negative correlation between $\phi _{4}$ and FCP. Therefore, it shows that it’s hard to draw conclusions about the underlying populations in studies employing simplified or averaged geometries that do not represent the evidently relevant anatomical features or natural combinations of these features. The same principle can be applied to investigate the influence of naturally occurring-anatomical features combinations on stress and strain levels and distributions in implanted models rather than the natural ones used in this study. Therefore, using this approach can improve the design process of spinal implants.

Furthermore, a relatively limited number of subject-specific training samples can be used to generate a large number of plausible models. It is also shown that a small number of important SMs can be used to generate much larger synthetic cohorts that represent the most important shape variations. This will significantly reduce the dimensionality, allowing a more efficient sampling of the shape space and increasing the efficiency of device design and testing during optimisation studies and in-silico trials, respectively.

A limitation of the presented FEMs is the use of only axial compression loading. Applying other loading conditions involving bending or rotation motions would require the inclusion of ligaments. Nonetheless, the proper modelling of ligaments is a complex aspect that requires careful consideration of appropriate material models and pre-loads that are hard to measure and an experimental validation step that was not possible for the considered training models.

The FEMs utilised a set of homogeneous materials, as well as propagating the same material properties across all subjects. However, studies suggest a high inter-subject variability in material properties,^10^ as well as a correlation between shape and the spatial distribution of material properties.^24,25^ Therefore, the inclusion of spacial material properties in the parameterisation process is likely to produce more objectively realistic results.

A relatively small number of shape modes was used for generating synthetic subjects. Using more than 30 SMs (e.g., explaining 90% or 95% shape variation) would increase the SSM accuracy by 16% and 40%, respectively. It is also known that lower variance shape modes can be crucial in predicting an output, depending on the specific data being analysed.^21^ Nonetheless, increasing the number of shape modes means increasing the number of predictors to be considered in subsequent steps, which would have resulted in a larger minimum samples size, as it would have impacted the performance of the computationally intensive Shapley analysis. Therefore, a balance had to be struck between the number of included shape modes and the target statistical power of the study. Correlation and importance analyses showed a larger proportion of higher correlation coefficients and contributions in the first half of the included 30 SMs and a general trend of decreasing correlation and contribution with the increase of SM number. Therefore, excluding the remaining shape modes is believed to have little or no adverse impact on the importance analysis in this study.

As mentioned, 3-dimensional TPS is computationally intensive. Hence, a balance had to be struck between the mesh warping quality and the required computation time, which required down-sampling the control points instead of using all points produced by the SSM. The mean and maximum RMSE across synthetic shapes due to mesh warping are 0.37 mm and 0.56, respectively. Figure 4S shows the distribution of the node-to-node Euclidean distances between the corresponding SSM-generated and TPS-generated shapes associated with the maximum RMSE. Errors due to mesh warping are much smaller than errors induced by the SSM itself. On the other hand, it was noticed, as seen in (Fig. 4S), that this error can reach relatively high local values. Unlike SSM-induced errors, the error distribution pattern here is unpredictable. In other words, such large local errors can misrepresent some structures that have a significant impact on FE outcomes of interest, as well as the sensitivity analysis in this case. Therefore, a more efficient method for selecting appropriate control points should be considered to minimise the impact of mesh warping errors.

A relatively large sample size of 500 synthetic FEMs was generated and analysed. Both the power and the uncertainty quantification analyses showed that the sample size was sufficient for the application at hand. Nonetheless, even with the use of highly efficient sampling approaches, such as the Latin Hypercube method, 500 samples cannot effectively sample the whole 30-dimensional space to cover the possible range of anatomies present in a target (i.e., training) cohort, not to mention the inclusion of spatially varying material properties, consideration of multiple loading scenarios, or abnormal anatomies. Therefore, achieving the full potential of synthetic cohorts for large-scale applications such as *in-silico* trials would require much larger sample sizes. While generating the shapes as point clouds from the SSM is not computationally demanding, the subsequent mesh warping and finite element modelling steps would not be practical. Although the use of faster mesh generation tools may be possible, the time required for running finite element models is a barrier that cannot be easily overcome. Therefore, we believe that taking full advantage of synthetic cohorts requires FE simulation acceleration strategies,^20,40^ or incorporating data-driven mechanical analysis approaches.^11,35^

Conventional clinical trials are limited in terms of subject availability, in addition to other limitations such as long study times and inability to apply testing conditions that may be considered unethical, in which case they are applied experimentally and/or computationally in very limited sample sizes and/or animal models in the pre-clinical testing stage. Therefore, *in silico* trials have been proposed as a viable solution to such limitations by rigorously testing devices in computational studies employing a large number of samples and under comprehensive boundary conditions.^36,41^ This type of studies would require a data-driven based approach to overcome the computational load limitations mentioned above. Therefore, a large number of samples (i.e., FEMs) is required for algorithm training as well as for conducting the *in silico* trial itself. The presented workflow may be used for generating such a large number of samples.

In conclusion, The proposed method can produce spinal samples that reflect the shape variability in a real cohort. Furthermore, although individual shape modes (SMs) are independent in the principal component space, each SM normally affects more than one anatomical feature in the Cartesian space. Hence, this parameterisation approach is able, by construction, to capture correlations between anatomical features in real populations and produce plausible new (sampled) geometries.

## Supplementary Information

Below is the link to the electronic supplementary material.

Electronic supplementary material 1 (PDF 1125 kb)

## Footnotes

1 Figure numbers appended with an ’S’ indicate those appearing in the supplementary materials
