# Correction: Contribution of Shape Features to Intradiscal Pressure and Facets Contact Pressure in L4/L5 FSUs: An *In-Silico* Study

**DOI:** 10.1007/s10439-023-03149-6

**Published:** 2023-01-27

**Authors:** Amin Kassab-Bachi, Nishant Ravikumar, Ruth K. Wilcox, Alejandro F. Frangi, Zeike A. Taylor

**Affiliations:** 1 Institute of Medical and Biological Engineering (iMBE), School of Mechanical Engineering, University of Leeds, Leeds, LS2 9JT UK; 2 Centre for Computational Imaging & Simulation Technologies in Biomedicine (CISTIB), School of Computing, University of Leeds, Leeds, LS2 9BW UK; 3 Leeds Institute of Cardiovascular and Metabolic Medicine (LICAMM), School of Medicine, University of Leeds, Leeds, LS2 9JT UK

**Correction to: Annals of Biomedical Engineering (2022) 51:174-188** 10.1007/s10439-022-03072-2

In the original article the descriptions of Figs. 7a and 7b were transposed in the figure caption. The corrected Fig. 7 caption follows:

Figure 7. Individual shape mode percentage of contribution to FCP (a) and IDP (b).

