# Supplementary material for: Contribution of Shape Features to Intradiscal Pressure and Facets Contact Pressure in L4/L5 FSUs: An In-Silico Study
Source: Ann Biomed Eng. 2022 Sep 14;51(1):174–88. doi: 10.1007/s10439-022-03072-2 (PMC9831962; doi:10.1007/s10439-022-03072-2)
Supplement: Supplementary file 1 — Electronic supplementary material 1 (PDF 1125 kb) [file 10439_2022_3072_MOESM1_ESM.pdf]

Contribution of shape features to intradiscal pressure and facets contact pressure in L4/L5 FSUs: An *in-silico* study

Amin Kassab-Bachi[1,2], Nishant Ravikumar[2], Ruth K. Wilcox[1], Alejandro F. Frangi[2,3], and Zeike A. Taylor[1,2]

[1]Institute of Medical and Biological Engineering (iMBE), School of Mechanical Engineering, University of Leeds, Leeds, LS2 9JT, United Kingdom.
[2]Centre for Computational Imaging & Simulation Technologies in Biomedicine (CISTIB), School of Computing, University of Leeds, Leeds, LS2 9BW, United Kingdom.
[3]Leeds Institute of Cardiovascular and Metabolic Medicine (LICAMM), School of Medicine, University of Leeds, Leeds, LS2 9JT, United Kingdom.

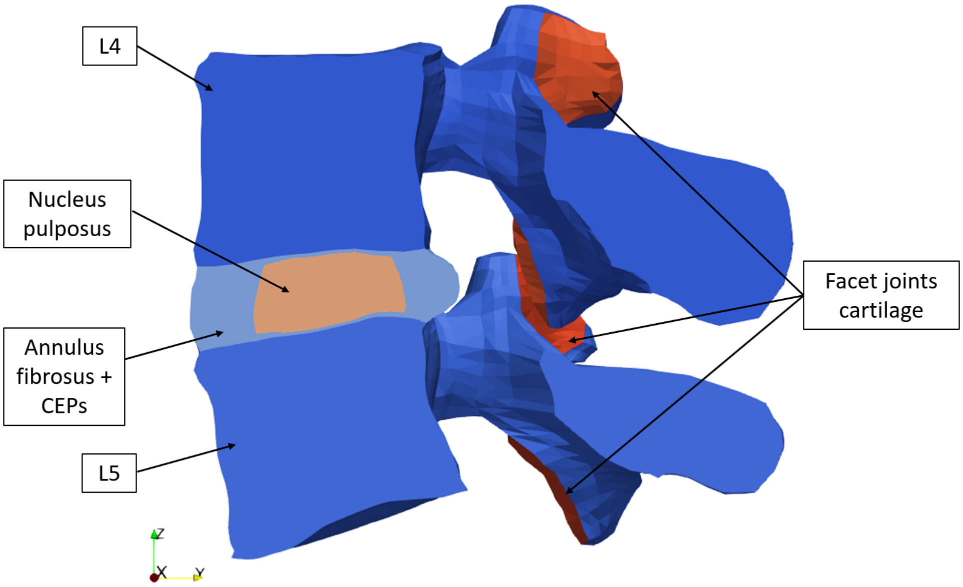

Figure 1S: Cross section view showing the distinct mesh regions of an L4/L5 segment geometry extracted from the MySpine database.

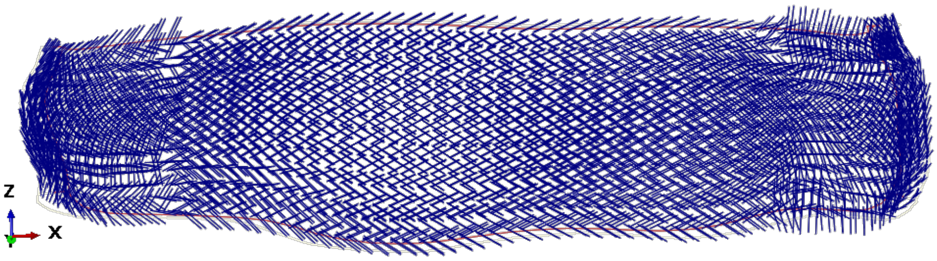

(a)

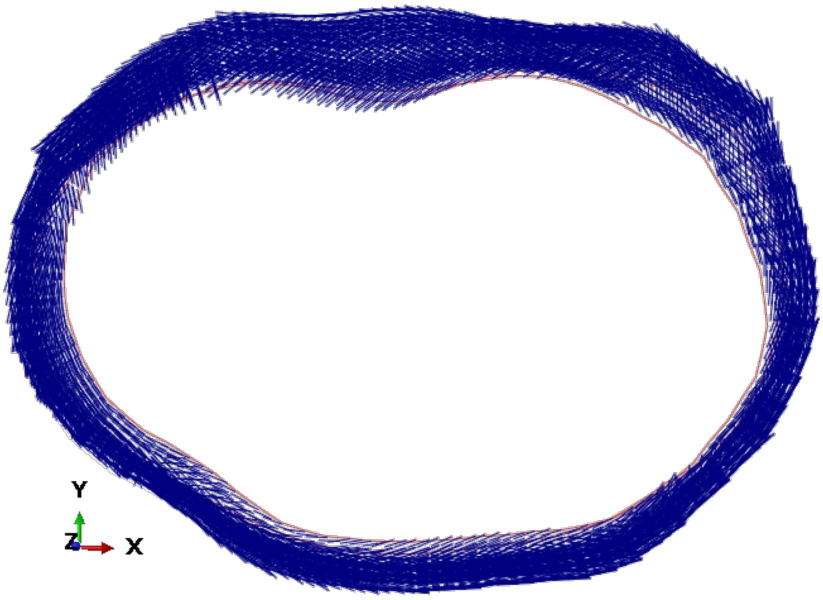

(b)

Figure 2S: Visualisation of the alternating ±30° fibre orientation in the two outer layers of the annulus fibrosus in frontal view (a) and top view (b).

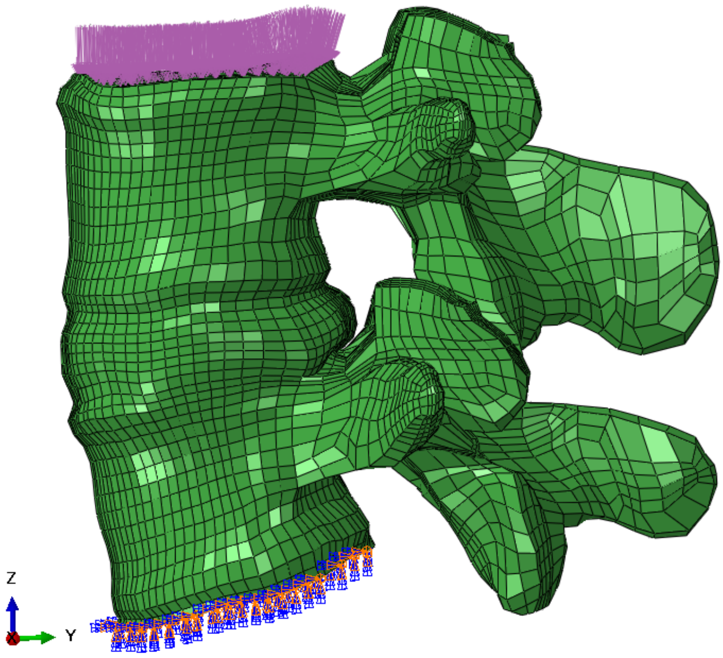

Figure 3S: Visualisation of the loading and boundary condition applied to the L4/L5 segments. The purple arrows represent a uniformly distributed pressure. The bottom of the segment was fully fixed as indicated by the blue/orange markers.

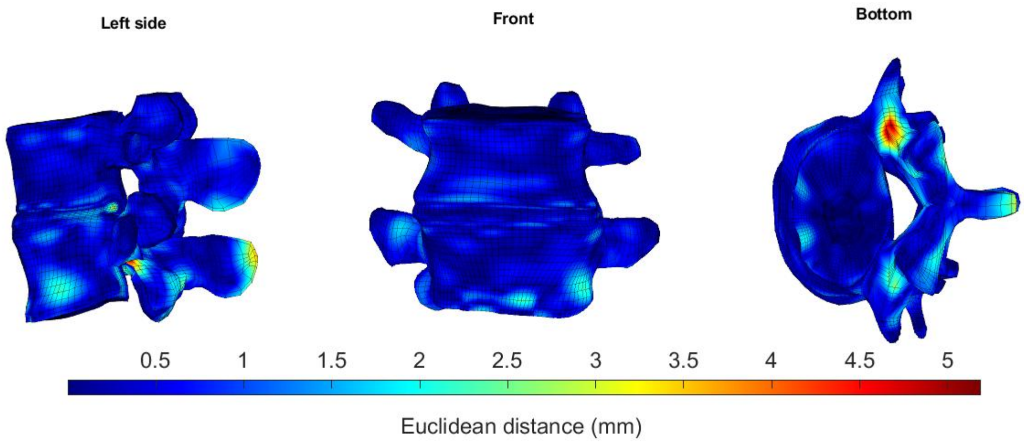

Figure 4S: The TPS-generated surface associated with the largest mesh warping error across the synthetic shapes. The heatmap represents the Euclidean distance between the SSM-generated and TPS-generated surfaces and shows high localised errors.
